# Supplementary material for: Subgiants in NGC 188 reveal that rotationally induced mixing creates the main sequence Li-Dip
Source: Nat Commun. 2025 Nov 4;16:9729. doi: 10.1038/s41467-025-64724-0 (PMC12586687; doi:10.1038/s41467-025-64724-0)
Supplement: Supplementary file 1 — Description of Additional Supplementary Files [file 41467_2025_64724_MOESM1_ESM.pdf]

Description of Additional Supplementary Files: Subgiants in NGC 188 Reveal that Rotationally Induced Mixing Creates the Main Sequence Li-Dip

Qinghui Sun[1,2*], Constantine P. Deliyannis[3], Barbara J. Anthony-Twarog[4], Bruce A. Twarog[4], Xiao-Tian Xu[5, 6], Aaron Steinhauer[7], Jeremy R. King[8]

[1*]Tsung-Dao Lee Institute, Shanghai Jiao Tong University, Shanghai, 201210, China.
[2*]Department of Astronomy, Tsinghua University, Beijing, 100084, China.
[3]Department of Astronomy, Indiana University, 727 East 3rd Street, Bloomington, 47408, IN, USA.
[4]Department of Physics and Astronomy, University of Kansas, 1251 Wescoe Hall Dr., Lawrence, 66045, KS, USA.
[5]School of Astronomy and Space Science, Nanjing University, Nanjing, 210093, Jiangsu, China.
[6]Argelander-Institut für Astronomie, Universität Bonn, Auf dem Hügel 71, Bonn, 53121, Germany.
[7]Department of Physics and Astronomy, State University of New York, Geneso, 14454, NY, USA.
[8]Department of Physics and Astronomy, Clemson University, 118 Kinard Laboratory, Clemson, 29634-0978, SC, USA.

*Corresponding author(s). E-mail(s): qinghuisun@sjtu.edu.cn;

File Name: Supplementary Data 1
Description: The table contains stellar parameters and lithium abundances for stars in NGC 188. Columns include the Platais ID from [1], coordinates (J2000; from [2]), V magnitude, $B-V$ color and its uncertainty (eB-V), $T_{\rm eff}$ and uncertainty (eTeff), surface gravity (log g), microturbulence velocity (Vt), projected rotational

velocity (VROT) and uncertainty (eVROT), radial velocity (VRAD) and uncertainty (eVRAD), membership classifications from [3], final membership status, lithium abundance A(Li) and uncertainty (eA(Li)), detection flag ("yes" for detection, "no" for non-detection), signal-to-noise ratio (S/N), and comments.

File Name: Supplementary Data 2
Description: Supplementary Table 2 presents radial velocity measurements and membership classifications for NGC 188 stars. It includes the Platais ID, V magnitude, $B-V$ color, radial velocities (VRAD), uncertainties (eVRAD), and configuration labels across up to nine observing epochs (n1 to n9). The final column gives the adopted membership classification based on radial velocity variability: SM for single member, BM for binary member, and "?" for uncertain classification.

References

[1] Platais, I., Kozhurina-Platais, V., Mathieu, R.D., Girard, T.M., van Altena, W.F.: WIYN Open Cluster Study. XVII. Astrometry and Membership to V=21 in NGC 188. The Astronomical Journal **126**(6), 2922–2935 (2003) https://doi.org/10.1086/379677 arXiv:astro-ph/0309749 [astro-ph]

[2] Gaia Collaboration, Brown, A.G.A., Vallenari, A., Prusti, T., de Bruijne, J.H.J., Babusiaux, C., Bailer-Jones, C.A.L., Biermann, M., Evans, D.W., Eyer, L., Jansen, F., Jordi, C., Klioner, S.A., Lammers, U., Lindegren, L., Luri, X., Mignard, F., Panem, C., Pourbaix, D., Randich, S., Sartoretti, P., Siddiqui, H.I., Soubiran, C., van Leeuwen, F., Walton, N.A., Arenou, F., Bastian, U., Cropper, M., Drimmel, R., Katz, D., Lattanzi, M.G., Bakker, J., Cacciari, C., Castañeda, J., Chaoul, L., Cheek, N., De Angeli, F., Fabricius, C., Guerra, R., Holl, B., Masana, E., Messineo, R., Mowlavi, N., Nienartowicz, K., Panuzzo, P., Portell, J., Riello, M., Seabroke, G.M., Tanga, P., Thévenin, F., Gracia-Abril, G., Comoretto, G., Garcia-Reinaldos, M., Teyssier, D., Altmann, M., Andrae, R., Audard, M., Bellas-Velidis, I., Benson, K., Berthier, J., Blomme, R., Burgess, P., Busso, G., Carry, B., Cellino, A., Clementini, G., Clotet, M., Creevey, O., Davidson, M., De Ridder, J., Delchambre, L., Dell'Oro, A., Ducourant, C., Fernández-Hernández, J., Fouesneau, M., Frémat, Y., Galluccio, L., García-Torres, M., González-Núñez, J., González-Vidal, J.J., Gosset, E., Guy, L.P., Halbwachs, J.-L., Hambly, N.C., Harrison, D.L., Hernández, J., Hestroffer, D., Hodgkin, S.T., Hutton, A., Jasniewicz, G., Jean-Antoine-Piccolo, A., Jordan, S., Korn, A.J., Krone-Martins, A., Lanzafame, A.C., Lebzelter, T., Löffler, W., Manteiga, M., Marrese, P.M., Martín-Fleitas, J.M., Moitinho, A., Mora, A., Muinonen, K., Osinde, J., Pancino, E., Pauwels, T., Petit, J.-M., Recio-Blanco, A., Richards, P.J., Rimoldini, L., Robin, A.C., Sarro, L.M., Siopis, C., Smith, M., Sozzetti, A., Süveges, M., Torra, J., van Reeven, W., Abbas, U., Abreu Aramburu, A., Accart, S., Aerts, C., Altavilla, G., Álvarez, M.A., Alvarez, R., Alves, J., Anderson, R.I., Andrei, A.H., Anglada Varela, E., Antiche, E., Antoja, T., Arcay, B., Astraatmadja, T.L., Bach, N., Baker, S.G., Balaguer-Núñez, L., Balm, P., Barache, C., Barata, C., Barbato, D., Barblan, F., Barklem, P.S., Barrado, D., Barros, M., Barstow, M.A., Bartholomé Muñoz, S.,

Bassilana, J.-L., Becciani, U., Bellazzini, M., Berihuete, A., Bertone, S., Bianchi, L., Bienaymé, O., Blanco-Cuaresma, S., Boch, T., Boeche, C., Bombrun, A., Borrachero, R., Bossini, D., Bouquillon, S., Bourda, G., Bragaglia, A., Bramante, L., Breddels, M.A., Bressan, A., Brouillet, N., Brüsemeister, T., Brugaletta, E., Bucciarelli, B., Burlacu, A., Busonero, D., Butkevich, A.G., Buzzi, R., Caffau, E., Cancelliere, R., Cannizzaro, G., Cantat-Gaudin, T., Carballo, R., Carlucci, T., Carrasco, J.M., Casamiquela, L., Castellani, M., Castro-Ginard, A., Charlot, P., Chemin, L., Chiavassa, A., Cocozza, G., Costigan, G., Cowell, S., Crifo, F., Crosta, M., Crowley, C., Cuypers, J., Dafonte, C., Damerdji, Y., Dapergolas, A., David, P., David, M., de Laverny, P., De Luise, F.: Gaia Data Release 2. Summary of the contents and survey properties. Astronomy and Astrophysics **616**, 1 (2018) https://doi.org/10.1051/0004-6361/201833051 arXiv:1804.09365 [astro-ph.GA]

[3] Geller, A.M., Mathieu, R.D., Harris, H.C., McClure, R.D.: WIYN Open Cluster Study. XXXII. Stellar Radial Velocities in the Old Open Cluster NGC 188. The Astronomical Journal **135**(6), 2264–2278 (2008) https://doi.org/10.1088/0004-6256/135/6/2264 arXiv:1512.04983 [astro-ph.SR]
